# Book Review

**Published:** 1949-04

**Authors:** 


### Reviews of Books

**Diseases of the Ear, Nose and Throat.** By D. G. Carruthers, F.R.A.C.S. Second Edition. Pp. 344. Illustrated. Bristol: John Wright & Sons Ltd. Price 25s.—This book is disappointing: parts are excellent, but quite a large proportion is inadequate. The illustrations are all good, but too many are devoted to instruments and operations. Indeed, too much space is given to operative technique; for example, twenty pages to tonsillectomy, almost as much as to the whole of "Larynx". X-rays of the sinuses are good: but the only other methods of diagnosing sinus disease that receive mention are the Victorian procedures of transillumination and proof-puncture, still apparently in use in the Antipodes—endoscopy, displacement, suction are not even mentioned. The first edition of the book was apparently well received in Australia, but we cannot recommended it to students or practitioners here.

**A Manual of Practical Obstetrics.** By O'Donel Browne, Litt.D., M.B., F.R.C.P., F.R.C.O.G. Second Edition. Pp. viii, 267. Illustrated. Bristol: John Wright & Sons Ltd. 1948. Price 35s.—This book sets out to be a manual of practical obstetrics, and those chapters dealing with the actual practice of obstetrics are good and helpful to the student. It wastes pages on the corpus luteum of menstruation and pregnancy, the development of the ovum, and the anatomy of the pelvis ; these being dealt with in such brevity that they might with advantage have been omitted. The chapter on the diagnosis of pregnancy lays emphasis on Hegar's sign. In this country students are taught never to elicit it because of the danger of producing abortion. Many of the techniques advocated have been condemned by British obstetricians : e.g. induction of labour by bougies, plugging the uterus for post-partum hæmorrhage, and traction on the child's body in breech delivery. The subject-matter dealt with in the chapter on X-rays is excellent, but could be improved by showing plates of X-ray pelvimetry. The book is well set out, and arranged for rapid consultation by a student in an emergency, but its teaching is in many aspects opposed to that of British schools.

**Practical Orthoptics in the Treatment of Squint.** Third Edition. By T. KEITH LYLE, M.D., M.Ch., M.R.C.P., F.R.C.S., and SYLVIA JACKSON, S.R.N., D.B.O. Pp. xii, 271. Illustrated. London: H. K. Lewis & Co. Ltd. 1949. Price 35s.—Though perhaps primarily addressed to students who will be sitting for the Diploma of the British Orthoptic Board, this book will, in fact, appeal to all interested in the treatment of squint cases. The theory and practical management of orthoptic cases, the potentialities and limitations of training, are clearly set forth. Latent and paralytic strabismus is discussed at length. This section of the book contains many valuable illustrative case-histories from the authors' immense knowledge and practical experience in this branch of ophthalmological practice. The subject-matter has been brought completely up to date and incorporates all recent advances in orthoptics and the treatment of strabismus generally. The presentation is logical and extraordinarily lucid. The many illustrations, diagrams and charts permit of no misinterpretations. The book is complete with bibliography, glossary, and a full and accurate index, faultless in production, and worthy of the highest praise.

**Malignant Disease and Its Treatment by Radium.** Vols. I and II. By SIR STANFORD CADE, K.B.E., C.B., F.R.C.S., M.R.C.P. Second Edition. Pp. xi, 383 ; xi, 430. Illustrated. Bristol : John Wright & Sons Ltd. 1948–49. Price 52s. 6d. per volume.—The first two volumes of the four which comprise the second edition of this work have now been published. The first volume contains Parts I and II of the work ; the third and main part being formed by the remaining three volumes. In Part I are surveyed the properties of radium, its radiations, and the techniques for applying those radiations to the treatment of malignant diseases. Part II contains the description of the biological effects of irradiation. The physical section covers its ground adequately and contains chapters by three of the foremost physicists. The biological section presents a present-day picture of the effects of radiation, mainly from a clinician's point of view. Full reference is made to the classic researches of past workers, while modern experimental work is described in a chapter by Dr. F. G. Spear. The whole volume is a store of information, and contains an extensive bibliography. There are few aspects of the subject upon which specialists will fail to obtain guidance, while a general surgeon or physician should also find much of interest. The second volume covers approximately the field which is of interest to the ear, nose and throat surgeon. Malignant disease of the thyroid and salivary gland have been included in its scope. It begins the main part of a work which will be an unrivalled exposition of the whole field of radium treatment. This is an essential volume for the radiotherapist. Nowhere else will he find so authoritatively detailed and practical an account of the pathology, symptoms and signs of malignant growths, and their treatment with radium. Every surgeon who has to deal with these cases in any number should have access to the book. The study of it must inevitably improve his appreciation of the respective uses of irradiation and surgery in the treatment of malignancy. The radiation therapist using this work may be regretful that, as the title indicates, the emphasis is on radium treatment, which the author tends to favour to the exclusion of X-ray therapy. But it may be remembered that in some of the foremost radiotherapy departments in the country small field X-ray therapy is used successfully where the author prescribes teleradium.

**Introduction to Public Health.** By E. W. Caryl Thomas, M.D., B.Sc., D.P.H., Barrister-at-Law. Pp. 271. Bristol: John Wright & Sons Ltd. 1949. Price 15s.—This book has been written since the National Health Service Act came into force on July 5th, 1948. In ordinary times it is very difficult to obtain an up-to-date book on public health. It is more than ever necessary in view of the Health Act and, indeed, a new approach is essential in the circumstances. This book meets these requirements very well. The writer points out that it is limited in scope, for, as its title indicates, it is an *introduction* to public health. "It is an amplification of the notes of lectures on hygiene and public health given to medical students." The approach through the historic background makes the subject more interesting. It can be strongly recommended, not only to medical students working for their final examination, but also to student sanitary inspectors and health visitors and, indeed, to doctors in general practice who want to be brought up to date on this subject.

**Clinical Examination of the Nervous System.** Ninth Edition. By G. H. Monrad-Krohn, M.D., F.R.C.P. Pp. xx, 459. Illustrated. London: H. K. Lewis & Co. Ltd. 1948. Price 16s.—This book achieves admirably its purpose of presenting the clinical facts of neurology in a clear and concise form. It is the more commendable because the text is the author's own writing in exemplary English. The book is reliable and up to date. Although emphasis is rightly placed on the importance of the clinical method, appropriate space is given to the newer radiographic methods of investigation. The production is excellent and in this, the ninth, edition art-paper has been used throughout. Illustrations of high quality abound; they are drawn from the author's superb collection. The book has long been deservedly popular with students; and the practising neurologist will find useful information in these pages.

**Cum Notitia.** By D. A. ALEXANDER, M.B., Ch.B. Pp. 394. Bristol: John Wright & Sons Ltd. 1949. Price 12s. 6d.—This volume consists of a series of short, clinical notes of cases observed by the author during a long life in general practice. It is full of interesting observations and comment, presented in a highly individualistic style—which, indeed, at times renders the meaning somewhat obscure. "I," "we" and "one," or two different spellings of the same word in adjacent lines, suggest that more care might have been given to editing and proof reading.

**Zinc Ions in Ear, Nose and Throat Work.** By A. R. FRIEL, M.D., F.R.C.S. Pp. 60. Illustrated. Bristol: John Wright & Sons Ltd. 1948. 5s. 6d.—This book, which is in the main an epitome of the author's *Notes on Chronic Otohorrœa* published twenty years ago, suffers from the condensation both in style of the text and in the illustrations, the latter falling far below the standard of the previous volume. It is primarily written for medical officers in charge of school aural clinics and their assistants, and describes the principles and application of this method of treatment. Ionization is spectacular and mysterious, and appeals strongly to lay committees: it has been revived five or six times since it was abandoned in 1890. The end results are not superior to those of other modern methods. The present extension of the work to the wider field of the nose and throat describes methods which are time-consuming and unlikely to displace better-known surgical procedure.

